# PodNet: Pod real-time instance segmentation in pre-harvest soybean fields

**DOI:** 10.1016/j.plaphe.2025.100052

**Published:** 2025-05-19

**Authors:** Shuo Zhou, Qixin Sun, Ning Zhang, Xiujuan Chai, Tan Sun

**Affiliations:** a Agricultural Information Institute, Chinese Academy of Agricultural Sciences, Beijing, China; b Key Laboratory of Agricultural Big Data, Ministry of Agriculture and Rural Affairs, Beijing, China; c Zibo Institute for Digital Agriculture and Rural Research, Zibo, China

**Keywords:** Pre-harvest dataset, Soybean pod, Instance segmentation, High-throughput field phenotyping

## Abstract

Noninvasive analysis of pod phenotypic traits under field conditions is crucial for soybean breeding research. However, previous pod phenotyping studies focused on postharvest materials or were limited to indoor scenarios, failing to generalize to real-field environments. To address these issues, this paper employs an instance segmentation approach for the precise extraction of the pod area from multiplant RGB images in preharvest soybean fields. We first introduce a cost-effective workflow for constructing datasets of densely planted crop images with a uniform backdrop. Starting with video recording, high-quality static frames are collected by automatic selection. Then, a large vision model is explored to facilitate dense annotation and build a large-scale soybean dataset comprising 20k pod masks. Second, the pod instance segmentation model PodNet is developed based on the YOLOv8 architecture. We propose a novel hierarchical prototype aggregation strategy to fuse multiscale semantic features and a U-EMA prototype generation network to improve the model's perception performance for small objects. Comprehensive experiments suggest that lightweight PodNet achieves a superior mean average accuracy of 0.786 in the custom pod segmentation dataset. PodNet also performs competitively on in-field images without a backdrop and enables real-time inference on the edge computing platform. To the best of our knowledge, PodNet is the first pod instance segmentation model for preharvest fields. The low-cost and high-precision extraction of pods is not only a prerequisite for phenotypic analysis of the pod organs but also constitutes an important foundation in conducting cross-scale phenotyping from whole-plant to seed levels.

## 1 Introduction

Soybean (*Glycine max*) is the most important legume crop grown worldwide because of its protein and oil content. To improve soybean varieties and increase their yield and nutritional content, extensive research on soybean breeding has been conducted over the years from a genotype-phenotype perspective. Among the many phenotypic characteristics of soybean plants, the pod is one of the pivotal organs that directly influences both yield and quality. In soybean phenotyping studies, pod morphology serves as a key indicator to discern the characteristics of the plant. For example, the dimensions of pods play a crucial role in determining nutrient partitioning within individual seeds, thus impacting 100-seed weight and overall seed quality [1]. The number of pods per plant and the total pod count are significant parameters for assessing soybean yield potential [2]. Pod color is also associated with maturity attributes, providing valuable insights into harvest readiness [3]. The precise perception and evaluation of pods hold paramount importance in the effort to breed novel soybean cultivars with increased yield potential and superior quality. However, traditional phenotypic measurements are inherently labor intensive because of the massive sample volume and temporal continuous observations. The high demand for manual work not only limits the scale of experiments but also introduces potential subjective error.

**Pod phenotyping by deep learning.** To reduce the reliance on human labor while enhancing the accuracy and consistency of phenotypic data analysis, considerable scholarly efforts have been devoted to machine vision techniques and end-to-end solutions. Uzal et al. [4] first introduced an early convolutional neural network (CNN) to count the number of seeds per pod through an image classification paradigm. In this work, the deep learning approach exhibited substantial performance advantages over digital image processing (DIP) and conventional machine learning methodologies. Li et al. [5] released a dataset of 500 pod RGB images for the soybean seed counting task and proposed a CNN variant to output a density map of the seeds, followed by pixelwise summation. Li et al. [6] modified a CNN model [7] to segment stems, pods and nodes in images with a black background, and then, pixel-level phenotypic traits were calculated through PCA and DIP methods. In the literature [8], soybean plants were grown in pots and photographed from different views. The performance of several pod detection CNN models was compared, and leaf recognition was combined to predict soybean yield. Recently, as a visual algorithm framework with competitive overall performance and developability, the YOLO [9] architecture has been modified by many researchers for various organ phenotyping tasks in soybean crops [[10], [11], [12], [13], [14], [15], [16], [17], [18], [19], [20]]. These studies employed advanced deep learning methods to detect and classify soybean seed and pod traits with minimal human intervention in postharvest, potted, field and drone scenarios, thereby expediting the breeding and selection processes for high-yielding soybean cultivars. For low-throughput precise phenotyping [21,22], obtained point cloud data of a single soybean plant grown indoors via multiview stereo reconstruction or the neural radiance field method and then trained a 3D segmentation model to extract accurate pods and stem organs and their apparent parameters.

**Soybean pod synthetic dataset.** The aforementioned research on soybeans based on supervised deep learning typically requires the construction of task-specific labeled datasets for model training. However, traditional agricultural data collection is constrained by the seasonal and geographic nature of crops. The complexity of data organization and cleaning generally leads to high costs of assembling and annotating agricultural datasets. To alleviate the substantial demand for human labor in data annotation, a series of studies [[23], [24], [25], [26], [27]] pioneered a paradigm in which population pod datasets were synthesized from individual pod images with known annotations. They utilized images of soybean pods with a color-consistent background, applied DIP techniques to crop out individual pod regions into separate subimages, and then randomly distributed these single-pod subimages to compose new multipod images. Given that the boundaries of each pod in the synthesized images are known, manual annotation of the dataset is avoided, thereby significantly reducing the dataset construction costs. However, such studies are limited to images of postharvest materials captured in controlled laboratory settings and thus fail to be generalized to complex scenarios encountered in real-world conditions. In specific agricultural scenarios, supervised deep learning based on labeled data remains the primary method to achieve high performance under limited computing power. In a more pioneering approach [28], a synthetic digital twin of a plant field was simulated using the Unreal Engine 5 software. Large amounts of data were captured through the rendering system, reducing the need for manual labeling. The study claimed that the pipeline can be adapted to analyze soybean diseases.

**Soybean phenotyping in preharvest fields.** Among the studies that have been extended to field-based soybean plant phenotyping, some have used drone platforms to obtain close-up canopy images of soybeans in the field. One study [29] open-sourced a drone-acquired image dataset and trained a point-based counting network to detect pods. Among the many ground-based methods, Fu et al. [30] and Mathew et al. [31] shared a similar idea to extract the target plant row from the camera's field of view. A vehicle with depth cameras was manufactured to capture side-view RGBD images of soybean plants in preharvest fields. The depth frames are aligned with RGB images, from which foreground pixels of soybean plants are extracted using depth information. Fu et al. [30] designed an improved YOLOv5 model to perform multiclass object detection of pods with different numbers of seeds. Although the reported rate of missed detection of soybean pods reached 20 ​%, an additional quantity compensation model for correction is needed. Similarly, Mathew et al. [31] trained a YOLOv7 model to perform single class detection for the pods per plant counting task. However, the complexity of the procedures, the requirement for additional corrections, and the potential failure of depth cameras in outdoor environments prevent this research approach from becoming mainstream. In previous studies [32,33], RGB images were collected via a handheld camera in soybean breeding fields. Slim subimages of individual soybean plants were manually cropped and annotated to form a seed point dataset. Seed counting and distribution tasks were solved via the seed localization paradigm. However, the soybean plants in the fields are sparsely planted with large gaps between rows and columns, resulting in obvious differences between the foreground and background in the image. Therefore, the seed localization model trained on close-up single-plant images reported a significant performance loss in panoramic images containing multiple soybean plants. In the most recent research [34], fisheye cameras were mounted on a ground robot to capture soybean videos, and a seed detection model was trained to estimate soybean yield, fully demonstrating a technical solution for a robot equipped with visual deep learning algorithms to achieve field phenotyping analysis.

The demands of high-throughput phenotyping have spurred the innovation of visual solutions, facilitating more efficient phenotypic studies in soybean and other crops. However, the visual data used in these studies, real or synthetic, were collected primarily in laboratory settings, where postharvest soybean plant materials and individual pods were systematically positioned in a controlled imaging environment to obtain high-resolution, background-stable and target-predictable RGB images. In preharvest fields, imaging conditions vary greatly, and there is significant occlusion among plant branches and leaves. Visual methods developed on the basis of postharvest materials and indoor environments are incapable of achieving high-throughput field phenotyping. More importantly, the widely complied paradigm of object detection based on rectangular bounding boxes presents inherent flaws when applied to soybeans grown in preharvest fields. Owing to the density and orientation of mature pods, most bounding boxes in the detection results contain multiple pod targets, thereby affecting subsequent single-pod processing and statistical analysis. A practical and deployable solution must account for the condition of densely planted fields and minimize manual processing of visual data.

In conclusion, the issue of pod perception in preharvest fields has not been adequately addressed. Specifically, the challenge lies in developing real-world field datasets and the precise sensing of pods under complex field conditions, which is crucial for understanding pod growth dynamics and ultimately improving soybean yield. In this paper, we formulate the precise perception of soybean pods in fields as a pod instance segmentation task. The main contributions of this paper are as follows.

• A low-cost and efficient workflow for building in-field crop datasets is introduced. Videos are recorded, and high-quality frames are filtered automatically. A large vision model is then employed to assist in dense pixel-level annotation.
• A large-scale soybean dataset is built. It consists of side-view and whole-plant videos acquired by single-directional horizontal scanning in preharvest fields, from which 488 static images with a uniform backdrop are collected and 20k pod segmentation masks are labeled and open-sourced.
• PodNet, the first real-time field pod instance segmentation model, is developed. It incorporates the proposed hierarchical prototype aggregation strategy to fuse multiscale semantic features, and the U-EMA prototype generation network is tailored for small objects. PodNet also performs well on images without any background.
• The proposed pipeline is also a foundational framework for general phenotypic analyses. By processing multiplant soybean images, complete morphological information of the in-field plant population can be captured, facilitating the integration of other visual phenotypic traits and thus supporting more comprehensive crop phenotyping studies.

## 2 Materials and methods

### 2.1 Field soybean pod instance segmentation dataset

**Fig. 1:**
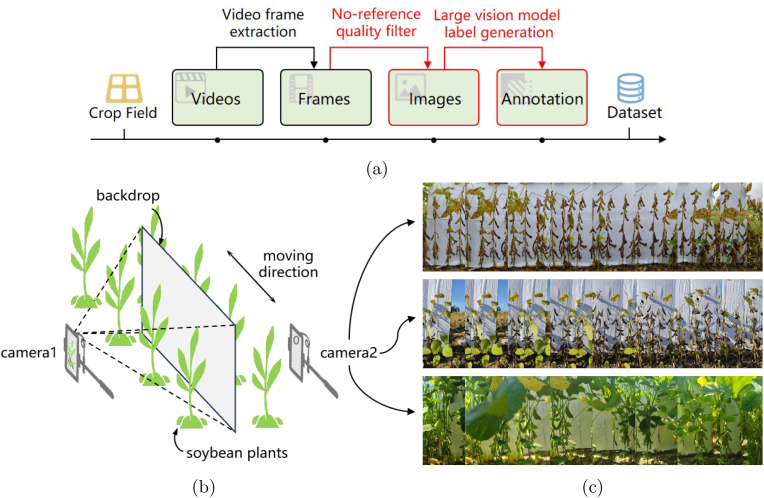
Field soybean data collection. (a) Workflow of field crop dataset construction. (b) Schematic diagram of video recording in preharvest fields. (c) Frame sequence of some captured videos.

In the supervised learning paradigm, large-scale image data and their corresponding annotations are indispensable. As introduced above, the predominant studies are based primarily on data from controlled environments such as laboratories, greenhouses, and expensive facilities, which naturally do not replicate the conditions in preharvest agricultural fields. Furthermore, the current mainstream pod perception methods based on object detection have inherent flaws with rectangular bounding boxes. Especially in densely planted field scenarios, designing datasets that support algorithms suitable for practical field applications is challenging. To address the problem of the high costs of acquiring agricultural data, this paper presents a feasible workflow to construct in-field image datasets with a uniform backdrop, which significantly reduces human labor costs from two aspects of the collection and annotation of agricultural visual data, as Fig. 1 (a) illustrates.

#### 2.1.1 Field soybean data collection

The data collection methodology applied in this study is expected to adhere to the following principles. (1) Ensure consistency between the training and testing domains, with a focus on aligning collected data closely with the potential application scenarios. (2) Noninvasive approaches should be applied to avoid any disruption to the ongoing scientific breeding process. (3) Capture the whole plant view rather than parts, thereby enabling concurrent acquisition of phenotypic information at both the plant and organ levels. However, many constraints exist in real-world environments. For example, soybean crops are densely planted, making it impractical to deploy large or multi-person-operation imaging equipment. Since the pod growth sites are concentrated on the main stem, overhead imaging is hindered by the leaves and upper pods, leading to obstructed views of the lower pods and substantial information loss.

Considering the limitations and workload required for data collection, this paper ultimately adopts the following workflow. (1) Data collection is conducted on soybean plants during the seed-filling and maturity stages for pod access. Images are collected from the side view of the target plant row, and a solid-color backdrop is used to separate the current row and eliminate distractions from the other rows in the camera's view. (2) To obtain as much diverse data as possible and reduce human labor costs, continuous video streams are recorded directly in fields to construct the original dataset. Video frame extraction and no-reference image quality assessment methods are used to obtain static images. (3) Smartphones are among the few electronic products whose screen brightness is sufficient for operation under sunny field conditions. Given that this paper exclusively utilizes RGB data, we select smartphone cameras as the data acquisition devices. The specific protocol of field data collection is illustrated in Fig. 1. For low cost, a white backdrop is fabricated simply by covering a cardboard sheet with white papers to serve as the separating tool for imaging. The long edge of the camera's field of view is maintained vertically and is aligned with the direction of soybean plant growth. To enhance efficiency, two cameras are positioned on either side of the backdrop, simultaneously capturing videos from two rows of soybeans. The camera view covers the whole target plant as much as possible. During video recording, image quality is assured by setting the resolution to no less than 1,280 ​× ​720 pixels and the frame rate to a minimum of 60 frames per second (FPS). Researchers hold the phone and move along the plant's row while recording the videos. Turning to the autozoom function of the lens can further reduce the manual workload during video acquisition. In situations where plants are excessively tall or row spacing is narrow, the smartphone's wide-angle lens is activated to facilitate comprehensive coverage.

**Fig. 2:**
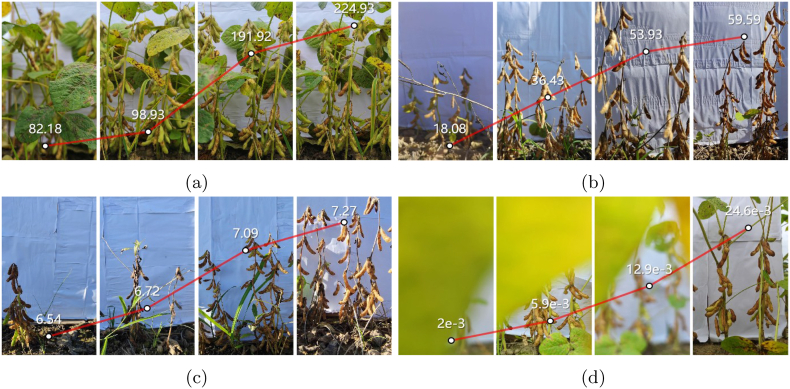
Example of image differences from the perspective of no-reference image quality assessment. (a) Image clarity. (b) Edge sharpness. (c) Image entropy. (d) GLCM energy.

**Why use backdrop in preharvest fields.** This paper sets up a solid-color backdrop in a preharvest field environment, which is necessary. As Fig. 2, Fig. 12 illustrate, in a densely planted field, the distance between rows of soybeans is so close that side-view imaging of plants without separators introduces distractions from neighboring rows in the background of the camera view. Although this situation does not hinder the establishment of the pure pod segmentation task, it complicates the cross-scale phenotypic analysis of the targeted row and single plant. Note that pod segmentation is not the ultimate goal of phenotypic studies. Rather, it serves as a foundation for the integrated phenotyping of pods with plants and other organs. Therefore, in the formulation of this problem, it is essential to guarantee the distinguishability of single-row soybean plants. Considering the analyzability of data, the use of only an RGB camera with a backdrop represents the most cost-effective and efficient method suitable for future research. Objectively, the backdrop slightly reduces the difficulty of the segmentation task, whereas the proposed methods also exhibit strong generalization ability for soybean images without any backdrop, including the images in datasets proposed in the literature [33]. The visualization examples and detailed explanations are discussed in Section 3.2.3.

#### 2.1.2 Field soybean image filtering

After video recording, frame extraction at an interval of one frame per second is employed to decompose the video streams into static images of soybean plants in preharvest fields. Owing to the inevitable problems of lens defocusing, blocked view, and nonsoybean targets when recording videos, not all image frames can be used to construct the dataset. Before the final manual check, no-reference image quality assessment (NR-IQA) methods are used to remove low-quality soybean images. NR-IQA assesses the distortion degree and sensing quality of an image solely based on the features extracted from its pixel information, enabling automatic evaluation of image quality without requiring reference images or ground truth. In this paper, four indicators are used to screen out unexpected frames. Please see Table 4 in Sec. A of the supplementary materials for a detailed definition. Image clarity and edge sharpness are important indicators used to measure an image's ability to represent details, and they are usually closely related to the quality level of the image perceived by human eyes. Image entropy can describe the uncertainty or randomness of information in an image and is generally believed that high-quality images have high complexity and diversity in terms of pixel distribution. The energy of the gray-level co-occurrence matrix (GLCM) is suitable for evaluating the uniformity of the image and the consistency of the texture, with a high energy value indicating that the image has strong texture consistency. The numerical differences in these four indicators for some example images are shown in Fig. 2. Threshold-based methods are used to filter out high-quality images based on the values of these indicators of each image. Specifically, in the first stage, we apply loose thresholding to all extracted frames to filter out the worst-quality images. Since the data were collected by different operators for different soybean varieties under different light conditions, the dataset showed regularity in the visual characteristics within each subset but showed obvious differences between subsets, as shown in Fig. 2. To ensure a balanced sample size while maximizing the diversity of the data domain, various NR-IQA methods and thresholds were tuned to different parts of the dataset. Therefore, among the high-quality images that are ultimately selected, each image may have undergone different methods and thresholds.

#### 2.1.3 Pod instance segmentation annotation

Given that the content of the images covers entire soybean plants, the pod targets are typically dense and small objects. Traditional manual annotation based entirely on human operation is time-consuming and labor-intensive. In this study, we delve into the exploration of annotation tools powered by the large vision model (LVM) for the creation of agricultural datasets. The Segment Anything model (SAM) is an LVM proposed by Ref. [35] that emerged amidst the surge of large language models. It is a sophisticated semantic segmentation model trained on an extensive dataset comprising approximately 11 million images and more than 1 billion pixel-level annotations. SAM can address zero-shot image segmentation well in a variety of contexts. Although foundation models have strong generalization capabilities, their deployment cost is high, and they cannot run in real time even on desktop-level GPUs. Therefore, it is not feasible to carry out foundation model development directly in agricultural scenarios. With a functional interface, the SAM can be used for prompt-based and interactive object mask generation. In this paper, we choose the open-source software X-AnyLabeling [36], which supports deploying pretrained SAM-like model checkpoints, to carry out instance segmentation annotation of the soybean pods within the images.

**Fig. 3:**
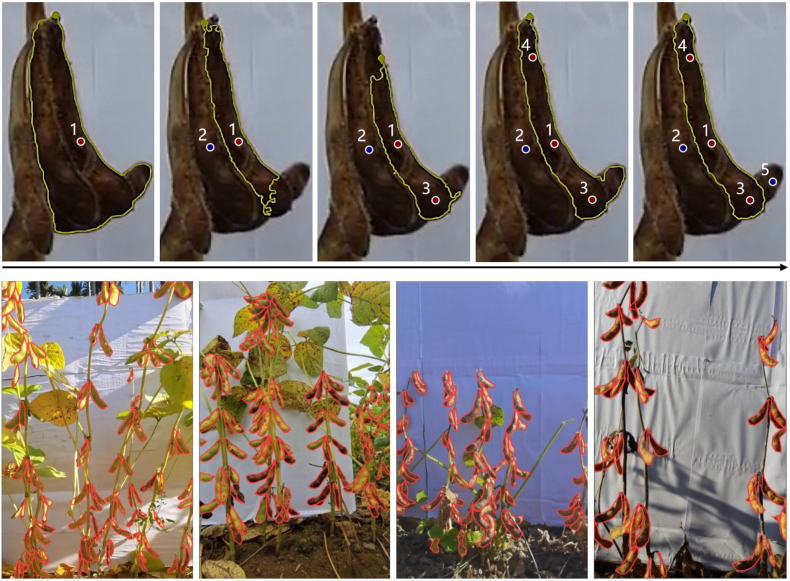
Pod instance segmentation annotation. Top: Instance mask generation by point prompts via the LVM. A red dot indicates that the region is to be added, and a blue dot indicates that the region is to be removed. Down: Visualization of some labeled images.

By using the LVM-based tool for mask generation of soybean pods, users can efficiently annotate images by clicking near the center of the target pod to provide prompt points. The SAM automatically generates a pixel-level candidate mask based on current prompt. For visually distinct and unobstructed pods, a single-point prompt might generate sufficiently high-quality mask labels. However, for pod clusters with complex backgrounds and mutual occlusions, the SAM may not produce the expected mask with only one click. To refine the candidate mask, additional point prompts can be continuously added. In Fig. 3, the red dots indicate that this area is inside the pod, the blue dots indicate that it is outside the excluded pod area, and the yellow polygon is the candidate mask generated in each step. Note that not all pod annotations can be completed with the assistance of the LVM, even if many points are added as prompts. In general, for pods that are densely stacked, have severe overlap, and have ambiguous boundaries, manual delineation of a closed polygon along the pod's edge is still indispensable.

### 2.2 Field soybean pod instance segmentation

Instance segmentation is a highly challenging task that integrates the advantages of object detection and semantic segmentation and enhances the granularity of image parsing. In this task, models are not only required to identify object categories but also to generate pixel-level segmentation masks for every individual instance within the image, thereby distinguishing between different objects of the same pod class. Owing to the pixelwise prediction involved and the concurrent handling of multiple objects, instance segmentation algorithms typically entail substantial computational complexity and memory occupation. Moreover, the use of the entire plant image as input makes the pod instance segmentation task a typical dense and small object perception problem. A key focus of this research is how to improve the model's segmentation quality with a lightweight model architecture.

**Fig. 4:**
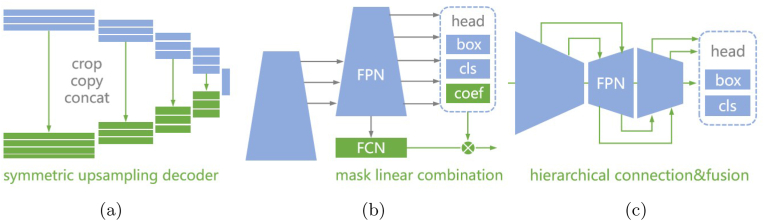
Architectures and key features of prominent instance segmentation models. The green part represents the core contribution of this method. (a) U-Net employs a symmetric encoder-decoder architecture with skip connections to preserve spatial information, making it highly effective for precise medical image segmentation. (b) YOLACT innovates with prototype mask generation and a linear combination of learnable coefficients, enabling real-time instance segmentation with high efficiency. (c) YOLOv8 enhances performance through hierarchical connection and fusion in its backbone, along with anchor-free detection and decoupled heads, for improved accuracy and speed.

**Prominent methods of object instance segmentation** In recent years, numerous excellent models focusing on segmentation tasks have emerged in the field of deep learning-based computer vision. U-Net [37] is a fully convolutional network (FCN) for image segmentation that employs a symmetric encoder-decoder structure and skip connections. As illustrated in Fig. 4 (a), a key characteristic of U-Net is its ability to preserve spatial information from the original input resolution, enabling the decoder to make full use of low-level features when reconstructing object boundaries, thereby producing precise pixel-level segmentation results. YOLACT (You Only Look At Coefficients) [7] is the first real-time instance segmentation framework that proposes the idea of prototype mask generation and learnable coefficients combination. In Fig. 4 (b), a small FCN produces a set of prototype masks, which represent partial or global visual features of different objects. Each target instance is represented by a set of prototypes and corresponding coefficients that determine the importance and combination of each prototype for the specific instance. YolactEdge [38] provides optimization and acceleration for inference of the YOLACT model, enabling real-time operation on edge computing devices with minor loss of performance. The YOLO (You Only Look Once) [9] series, as a leading architecture in the field of visual perception, has had a profound impact on both academia and industry due to its competitive performance. As depicted in Fig. 4 (c), YOLOv8 represents a milestone achievement in this series, incorporating innovations such as additional skip connections and partition operations in convolutional modules of the backbone network, a decoupled head structure for separate classification and bounding box prediction, and anchor-free detection. These enhancements have led to outstanding accuracy-efficiency trade-offs across a variety of vision tasks, making it particularly suitable for resource-constrained embedded devices.

#### 2.2.1 PodNet

**Fig. 5:**
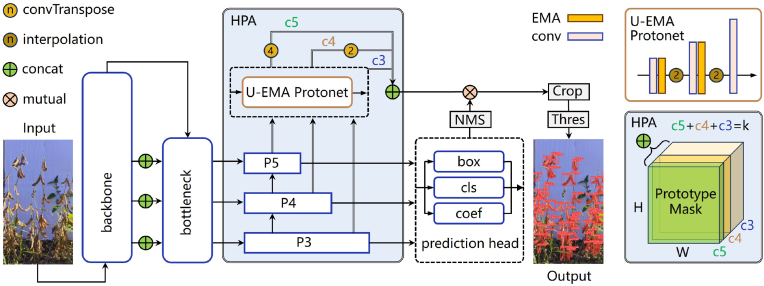
PodNet model architecture.

Taking advantage of prior research, this paper proposes the real-time field pod instance segmentation model PodNet. As Fig. 5 shows, PodNet is built upon the YOLOv8 backbone, with a decoder-encoder architecture to implement instance segmentation based on the principles of prototype mask generation and linear combination. To enhance the model's perception performance for dense and small pod targets, this study proposes the hierarchical prototype aggregation (HPA) strategy, which integrates multilevel feature semantic information, along with the U-decoder prototype generation network with EMA attention, which outputs more refined prototype masks.

#### 2.2.2 HPA: hierarchical prototype aggregation

In the original prototype linear combination of YOLACT, the prototype generation network (Protonet) relies solely on the P3 output from a specific scale of the feature pyramid network (FPN) to infer the k-channel prototype masks corresponding to an image, where k is a hyperparameter. Given that the P3 layer is relatively deep and has high spatial resolution, it typically provides the most detailed spatial information and is thus commonly selected as input for the Protonet to balance computational efficiency with segmentation performance. However, this approach does not fully leverage the rich semantic features contained in deeper layers of the bottleneck, leading to a lack of incorporation of higher-level semantic information into the prototype generation process.

**Fig. 6:**
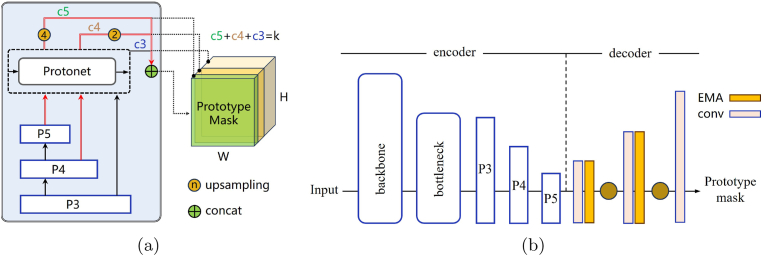
(a) Hierarchical Prototype Aggregation strategy. The red lines represent new pathways. (b) U-shaped architecture of PodNet.

To address these limitations, this study introduces the hierarchical prototype aggregation (HPA) strategy, which significantly expands and optimizes the I/O pipeline of Protonet. See Fig. 5. Unlike previous approaches that utilize only the high-resolution feature map P3, the HPA integrates multiple feature map levels from shallow to deep hidden layers within the FPN. Specifically, new pathways in Fig. 6 (a) are created by upsampling operations to restore higher resolutions and channel concatenation to combine different levels of features effectively. Owing to the nature of convolutional neural networks, the feature maps from P3 to P5 exhibit decreasing resolution and increasing channel numbers. Therefore, in the HPA scheme, the P5 and P4 layers undergo 4x and 2x upsampling, respectively, to match the resolution of the P3 layer. These three scales of feature maps are then concatenated along the channel dimension to form the final prototype mask that integrates multiscale and contextual information. This enhancement aims to comprehensively capture and integrate the different scale details and global context information carried by feature maps at various levels, enabling Protonet to learn more accurate and semantically rich prototype masks.

However, when the implementation of HPA to fuse multiscale feature maps is considered, new challenges arise. First, the total number of channels in the prototype mask feature maps needs to be determined, i.e., the k value. Second, the method for upsampling lower-resolution feature maps to enable their aggregation must be considered. Finally, how to allocate the contributions of the three scales of features along the channel dimension reasonably for optimal performance is also a question. To address these issues, this study presents a series of carefully designed ablation experiments that determine the optimal number of feature channels, upsampling methods, and contribution of each scale to the pod segmentation task. Details are provided in section 3.2.1.

#### 2.2.3 U-EMA: U-decoder with EMA attention

To facilitate the end-to-end acquisition of whole-plant and organ-level joint phenotypic information, the input to the proposed model in this study is the global soybean plant image. Thus, in addition to optimizing the mask construction mechanism in the prototype generation branch, we also modify the Protonet architecture to improve its segmentation performance in complex scenes. The core concept of the U-Net-like decoder-encoder design is to ensure that the model can produce high-resolution feature maps as close as possible to the original image size during inference, which is crucial for capturing fine structures and small objects. However, under the existing v8n-seg framework, the maximum input scale processed by its multiscale prediction module is only one-sixth the resolution of the original image, which undoubtedly limits the model's ability to perceive small-sized pod targets and their intricate details.

This paper introduces the U-EMA Protonet, a U-decoder Protonet that utilizes an efficient multiscale attention (EMA) [39] mechanism. The EMA introduces a cross-spatial learning approach and multiscale parallel subnetworks to establish both short- and long-range dependencies, thereby enhancing feature representation. A distinctive advantage of the EMA module lies in its ability to retain information on each channel while avoiding the side effects associated with dimensionality reduction, and it achieves computational efficiency through processing within two parallel branches utilizing 1x1 and 3x3 convolution kernels. Compared with other plug-and-play attention mechanisms, the EMA not only improves model performance on classification and detection tasks but also demonstrates significant parameter efficiency. Specifically, on benchmarks such as CIFAR-100 and MS COCO, the EMA module has facilitated higher Top-1 accuracy and mAP scores, all while maintaining lower model complexity. In addition, upsampling modules are incorporated within the original Protonet, which is capable of upscaling the effective resolution of intermediate feature maps to one quarter the size of the original image. From the perspective of the computing graph of the mask prototype, a U-shaped structure is formed, as depicted in Fig. 6 (b). This modification significantly enhances the model's discriminative ability and segmentation accuracy for pod objects and their complex structures at occluded edges. Despite the additional upsampling processes that increase the resolution of feature maps in the model's hidden layers, the U-EMA prototype mask generation network maintains real-time operational capabilities, achieving a balance between speed and precision.

## 3 Results

### 3.1 Field soybean pod instance segmentation dataset

**Fig. 7:**
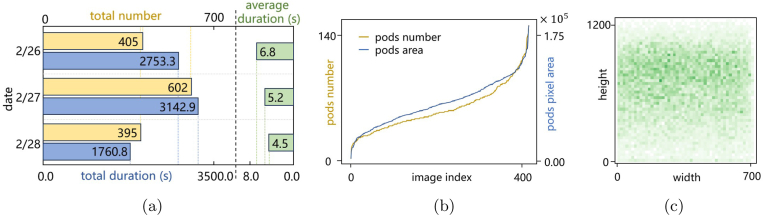
Pod annotation statistical analysis. (a) Number and duration (in seconds) of field soybean videos collected. (b) Distribution of the pod mask count and pixel area per image. (c) Pod bounding box center density map. This heatmap shows the location distribution of all the bounding box center points across the dataset.

Adopting a cost-effective and efficient data collection methodology, this study conducted data acquisition across various dates and times of the day, covering different light conditions and multiple varieties of preharvest soybeans. The use of continuous video frame extraction to collect image data from field pods has several significant advantages over the use of static pictures alone. In challenging field settings, minimizing site visits through efficient video recording strategies massively reduces human operational costs and time expenditure while maximizing data domain breadth. By embracing the variability in lighting change, wind influence, leaf occlusion and plant orientation, continuous video documentation ensures that the trained models are robust against many real-world complexities, thereby increasing their generalizability and practical utility. As illustrated in Fig. 7 (a), a total of 1,402 clips of consecutive videos, accumulating to more than 127 ​min of footage, are recorded. The average duration of all videos is 5.46 ​s per clip. In these videos, no soybean plants are recorded repeatedly.

Based on the NR-IQA evaluation indicators, a threshold-based method is then used to batch filter the image frames extracted from the videos. After discarding frames with unclear pods and severe occlusions, we obtain a total of more than 5k high-quality images from the raw video data, constituting a collection of in-field soybean images. Since the images in this dataset contain side-view images of whole soybean plants, they can be used for future studies, such as single-plant area detection, plant type analysis, soybean seed counting, and leaf disease identification. There are dense pod objects in one soybean image, and 500 images are initially selected randomly from the current images as candidates for the pod instance segmentation dataset. Although the NR-IQA methods automate image selection to a certain extent, pixel-based low-level feature evaluation cannot understand the semantic content of images, so we manually check them and finally determine 488 images as the dataset for annotation. A group of 406 images are randomly divided into a training set, 47 as a validation set and 35 as a test set. This pod dataset encompasses images of various soybean cultivars across different growth stages.

For dataset annotation, the Segment Anything (ViT-Base Quant) checkpoints are used. By employing the LVM tool, the average time required for instance, segmentation annotation of each image has been significantly reduced from 10.2 ​min to 6.5 ​min, thereby greatly improving the efficiency of manual annotation. The average pod number per image is more than 56, and the total number of pod objects is greater than 20k. Fig. 7 (c) shows that most of the pods are located in the upper center region of the image. The field soybean pod instance segmentation dataset is open sourced for the research community at https://github.com/Boatsure/PodNet.

### 3.2 Implementation and experiments of PodNet

Considering that instance segmentation is a computationally intensive task, this study selected the lightweight architecture YOLOv8-nano (v8n) as the baseline model for the development of PodNet. Model v8n has simplified module connections and competitive perception accuracy while significantly reducing the demand for hardware computational power. It aligns well with the resource-limited nature of agricultural equipment. By integrating the hierarchical prototype aggregation (HPA) and U-EMA Protonet methods into the v8n architecture, this study constructs a high-performance, lightweight model that is capable of performing small object perception tasks in field scenarios and meeting the low-power and low-cost deployment requirements of edge computing devices. The hyperparameter settings used in model training are listed in Table 5 of the supplemental materials, Sec. B. Note that not all the models support the hyperparameter items listed in the table.

To evaluate the performance of the models for the pod instance segmentation task, several key metrics are utilized. As depicted in Table 6 of the supplemental materials in Sec. B, TP represents the number of instances that are correctly identified by the model. FP denotes the number of instances that are incorrectly identified as positive by the model. FN represents the number of instances that were missed or incorrectly identified by the model. The accuracy, precision and recall metrics are related mainly to the model's performance in category judgment and cannot directly reflect the spatial positioning accuracy of masks. In the instance segmentation task, the IoU (intersection over union) is the core indicator used to measure the degree of overlap between the predicted mask and the ground truth mask. The mAP combines precision and recall and measures the model performance by the area under the precision-recall curve. Compared with a single accuracy metric, mAP@50 can more comprehensively reflect the classification and localization performance of the model. Moreover, compared with higher IoU thresholds, IoU ​= ​0.5 is a relatively loose standard and strikes a good balance between computational efficiency, practical application requirements, and model performance evaluation, especially for complex scenes or small targets. The accuracy tends to be affected by background pixels; too much precision may lead to missed detections, whereas too much recall may lead to false detections. Metric mAP@50 provides a compromise evaluation method, which makes it the mainstream metric used by the visual research community to evaluate instance segmentation models. The larger the values of these metrics during evaluation are, the higher the performance of the model.

#### 3.2.1 Experiments on the HPA component

The detailed results of the HPA experiments are shown in Table 1. Among the various quantitative metric analyses of models, the evaluation of model efficiency focuses primarily on GFLOPs (Giga Floating Point Operations), whereas the assessment of model performance typically focuses on the mAP@50 (mean average precision at an IoU threshold of 0.5) value. This study starts by evaluating the performance of several classic methods on the custom pod instance segmentation dataset.

**Table 1:** PodNet quantitative ablation experiments of HPA.

No.	model architecture	layers num	parameters/Million	GFLOPs	accuracy	recall	mAP @50	mAP @50-95
1	YOLACT-R50	–	–	–	–	–	0.673	–
2	YolactEdge-R50	–	–	–	–	–	0.603	–
3	YOLOv5n-seg	–	1.880	6.9	0.782	0.604	0.687	0.294
4	YOLOv5s-seg	–	7.398	25.7	0.808	0.656	0.738	0.335
5	YOLOv8n-seg	195	3.258	12.0	0.796	0.677	0.761	0.370

6	v8n-seg-nm16	195	3.110	9.3	0.782	0.680	0.749	0.362
7	v8n-seg-nm64	195	3.487	13.2	0.801	0.692	0.766	0.369
8	v8n-seg-nm128	195	4.113	16.1	0.793	0.684	0.756	0.366
9	v8n-seg-P34	163	2.161	11.1	0.778	0.690	0.754	0.370

10	v8n-seg-HPA1	213	3.586	13.2	0.809	0.689	0.769	0.372
11	v8n-seg-HPA2	213	3.586	13.2	0.809	0.692	0.771	0.376
12	v8n-seg-HPA3	213	5.592	13.3	0.804	0.669	0.759	0.370

13	PodNet-HPA1	216	3.537	12.3	0.807	0.709	0.780	0.401
14	PodNet-HPA3	216	3.537	12.4	0.799	0.704	0.779	0.399
**15**	**PodNet**	**216**	**3.537**	**12.4**	**0.805**	**0.709**	**0.786**	**0.398**

The following conclusions can be drawn from these experiments. (1) According to experiments No. 1–5 in Table 3, with the ongoing development of deep learning technology, the performance of the new algorithm models has consistently improved. Additionally, the number of parameters does not always correlate directly with model performance. For example, although YOLOv5s-seg has the highest number of parameters, its accuracy and recall do not significantly outperform those of v8n-seg, which has fewer parameters, and v8n-seg demonstrates superior performance in terms of the mean average precision (mAP). (2) Based on v8n-seg, experiments No. 6 through 8 investigate the number of prototype masks (nm), which is referred to as k in Fig. 7. The experimental results showed that the performance slightly improved when the nm was increased from 32 to 64 but decreased when it was further increased to 128, accompanied by an increase of more than 30 ​% in the number of GFLOPs. In summary, keeping the nm at 32, which is the baseline value, enables high accuracy while maintaining a lower computational load, making it a reasonable starting point for model improvements. (3) Furthermore, given that the YOLOv8 architecture employs multiscale feature fusion for object detection and instance segmentation, the P3, P4, and P5 scales in the model correspond to feature maps of different resolutions. Considering that all the pod targets in this study are small, No. 9 ​in Table 3 attempts to retain only the outputs from the P3 and P4 scales while removing the prediction head for P5. This resulted in a decline in performance. Although P5 has a lower spatial resolution, it captures contextual semantic information across the entire image range. Removing P5 may hinder the model's ability to infer the locations of small objects precisely from higher-level features. This also proves that deeper feature maps have a positive effect on model performance.

**Fig. 8:**
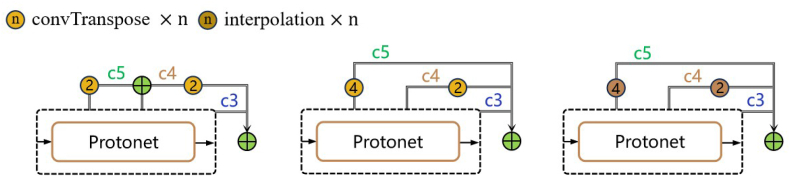
Different implementations for fusing multiscale feature maps in HPA. From left to right, they are cascaded transposed conv, parallel transposed conv and parallel interpolation.

In the design of HPA, there are multiple ways to implement the upsampling of the P4 and P5 feature maps. As Fig. 8 shows, (1) HPA1: cascaded transposed conv. First, P5 is upsampled via a transposed convolution (convT) layer by a factor of 2 and concatenated with the output of P4 to obtain the P45 feature map. Then, another convT operation is performed to upsample P45 by a factor of 2. (2) HPA2: parallel transposed conv. The output of P5 is upsampled via a convT module by a factor of 4, and the feature map of P4 is simultaneously upsampled via a 2x transposed convolution. These two outputs are then concatenated. (3) HPA3: parallel interpolation. The convT operations in HPA2 are replaced with nearest neighbor interpolation for upsampling. The ablation studies No. 10–15 suggest that the HPA architecture based on parallel transposed conv performs best in terms of both computational complexity and performance. Therefore, this method is ultimately applied in the PodNet model.

However, the contributions of the three scales P3, P4, and P5 to the 32 channels of the prototype mask output by Protonet also require experimental validation. As shown in Fig. 6a, the number of channels that compose the prototype mask from P3–P5 are denoted as *c*3, *c*4 and *c*5, respectively, and their relationship conforms to Equation (1). In a three-dimensional Cartesian coordinate system, Equation (1) defines a plane, and the combinations of channel numbers contributed by the three scales to the prototype mask can be considered as the solution space consisting of nonnegative integer points on this plane. Qualitatively, layers P3, P4, and P5 represent feature maps on different scales, where P3 has a higher spatial resolution and captures local details; P4 provides context information on a medium scale; and P5 contains global abstract semantic information.

(1) c3+c4+c5=k,k=32

In the original structure of Protonet, *c*3 ​= ​32. In the experiments shown in Table 2, the values of *c*4 and *c*5 increase gradually. The HPA strategy maximizes model performance when the channel counts are set to *c*3 ​= ​24, *c*4 ​= ​6 and *c*5 ​= ​2. A clear trend indicates that model performance steadily decreases with the reduction of *c*3 channels and the increase of *c*5 channels. In the HPA approach, the number of channels reflects the contribution of each scale to the final prototype mask. The large allocation of channels to P3 indicates that the model utilizes high-resolution features from this scale to generate pod segmentation masks. These high-resolution features are critical for capturing the fine contours and boundaries of pods. The P4 layer contributes only 6 channels, which provides medium-scale information that helps compensate for any shortcomings that might arise when dealing with larger or occluded pods using the P3 layer alone. The P5 layer, with only two channels, suggests that although the demand for the global context is relatively low in the pod segmentation task, a certain number of channels are retained to capture global background cues that may influence pod recognition. Channel distributions of 24, 6, and 2 are chosen to ensure optimal performance in pod segmentation and to make efficient use of computational resources. This aligns well with the requirements of field applications, where real-time processing and low computational complexity are crucial. In summary, these experiments demonstrate that the number of channels in the prototype mask output by the Protonet is 32, with the channel dimensions from different scales combined as 24 ​+ ​6 + 2.

**Table 2:** Contribution of the multi-scale feature on prototype mask composition.

c3	c4	c5	parameters/Million	GFLOPs	accuracy	recall	mAP @50	mAP @50-95
30	1	1	3.586	13.2	0.790	0.692	0.765	0.373
28	2	2	3.592	13.3	0.813	0.678	0.767	0.373
26	4	2	3.586	13.2	0.797	0.677	0.757	0.366
**24**	**6**	**2**	**3.586**	**13.2**	**0.809**	**0.692**	**0.771**	**0.376**
20	8	4	3.587	13.2	0.791	0.698	0.767	0.370
16	12	4	3.587	13.2	0.800	0.683	0.765	0.370
11	11	10	3.588	13.2	0.790	0.696	0.762	0.371
4	12	16	3.591	13.3	0.799	0.678	0.753	0.370

#### 3.2.2 Experiments on the U-EMA component

**Fig. 9:**
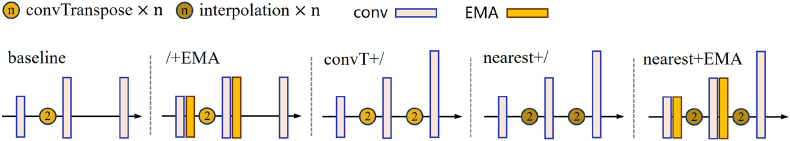
Candidate structures of the U-decoder Protonet.

As illustrated in Fig. 9 and Table 3, this paper validates the effectiveness of nearest neighbor interpolation upsampling and the EMA through ablation experiments, confirming their contribution to the U-decoder Protonet. Efficient multiscale attention (EMA) [39] reshapes the channel into batch dimensions to avoid a decrease in resolution and fuses the output feature maps of the two parallel subnetworks via a cross-spatial learning method. The addition of EMA after each convolutional layer enhances feature extraction capabilities with almost no increase in calculation.

**Table 3:** Ablation studies of U-EMA components.

U-decoder	EMA	layers num	parameters/Million	GFLOPs	accuracy	recall	mAP @50	mAP @50-95
–	–	195	3.258	12.0	0.796	0.677	0.761	0.370
–	*✓*	211	3.258	12.1	0.799	0.696	0.766	0.374
*✓*(convT)	–	196	3.274	15.6	0.808	0.703	0.775	0.398
*✓*(nearest)	–	196	3.242	11.5	0.816	0.697	0.783	0.400
*✓*(nearest)	*✓*	213	3.537	12.4	0.805	0.709	**0.786**	0.398

The results of the experiments conducted with the HPA and U-EMA Protonet, as shown in Tables 1 and 3, indicate that using transposed convolutions at the encoder stage of the model yields the best results, whereas nearest neighbor interpolation, as an upsampling method, performs optimally in the decoder stage. Transposed convolution, which serves as the inverse operation of conventional convolution, can restore and augment the spatial dimensions of feature maps. Its computational complexity is high, as it involves a substantial number of multiplication and addition operations. Nearest neighbor interpolation is a straightforward upscaling technique that generates higher-resolution images by duplicating the values of the nearest pixels. As it primarily involves indexing and copying operations with virtually no multiplication or addition operations, its theoretical number of floating-point operations (FLOPs) is negligible. In the bottleneck of the network, HPA facilitates the fusion of multiscale features by upsampling feature maps P3, P4 and P5 to an identical resolution and concatenating them. Transposed convolutions not only expand the spatial dimensions of the feature maps but also introduce additional learnable parameters during the upsampling process. This allows the model to effectively combine features at different scales through learned patterns, which is highly beneficial for capturing the representational characteristics of pods at various resolutions. In Protonet, the feature maps have already undergone multiple fuse operations prior to the decoder. At this point, further refinement through upsampling may yield diminishing returns in terms of overall segmentation accuracy, while maintaining model lightness and real-time performance becomes more critical. Using nearest neighbor interpolation avoids introducing additional parameters and computational complexity, which is beneficial for preserving model inference speed. Additionally, compared with kernel-based convolution operations, nearest-neighbor interpolation does not excessively smooth the original features, allowing for better preservation of edge and detail information in the higher-level feature maps.

In summary, transposed convolutions and nearest-neighbor interpolations leverage their respective strengths at different stages of the PodNet model, contributing to its high-performance instance segmentation of pods in preharvest fields. As an upsampling operation, transposed convolution effectively merges multiscale features and restores spatial resolution at the bottleneck stage, whereas nearest neighbor interpolation rapidly performs resizing at the decoder stage. The combination of these two techniques at different model stages enables PodNet to achieve high segmentation accuracy while maintaining real-time performance.

#### 3.2.3 PodNet inference results

**Fig. 10:**
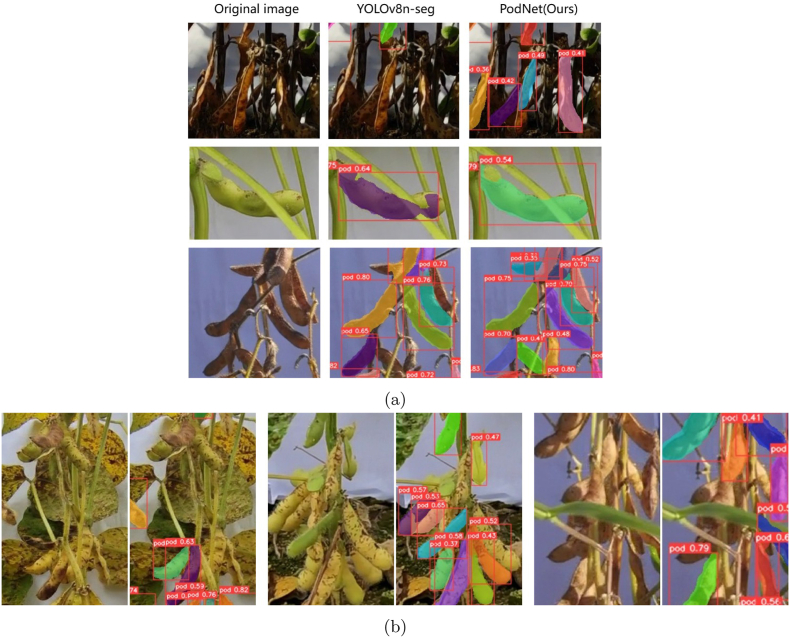
Qualitative comparison of instance segmentation results. Masks of different colors represent different segmented instances of soybean pods. The boxes indicate the minimum bounding rectangles of the segmented masks. The white numbers in the top-left corner of each red bounding box represent the confidence scores of the corresponding mask. (a) Improving cases of PodNet segmentation compared with YOLOv8n-seg. (b) Failure cases of PodNet segmentation. For each pair of images, the left image is the original image, and the right image is the PodNet segmentation result.

**Fig. 11:**
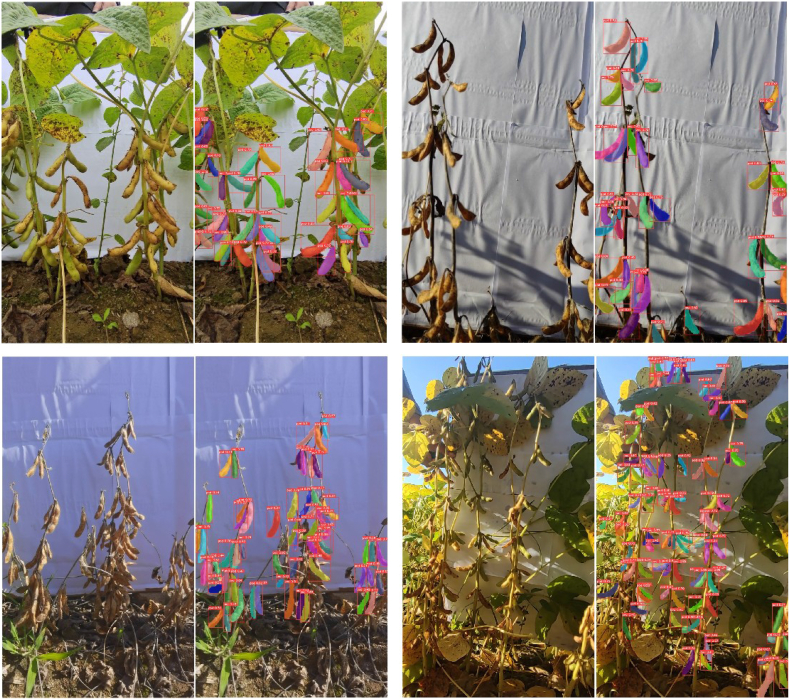
Visualization of several PodNet inference results. For each pair of images, the left image is the original image, and the right image is the PodNet segmentation result.

**Fig. 12:**
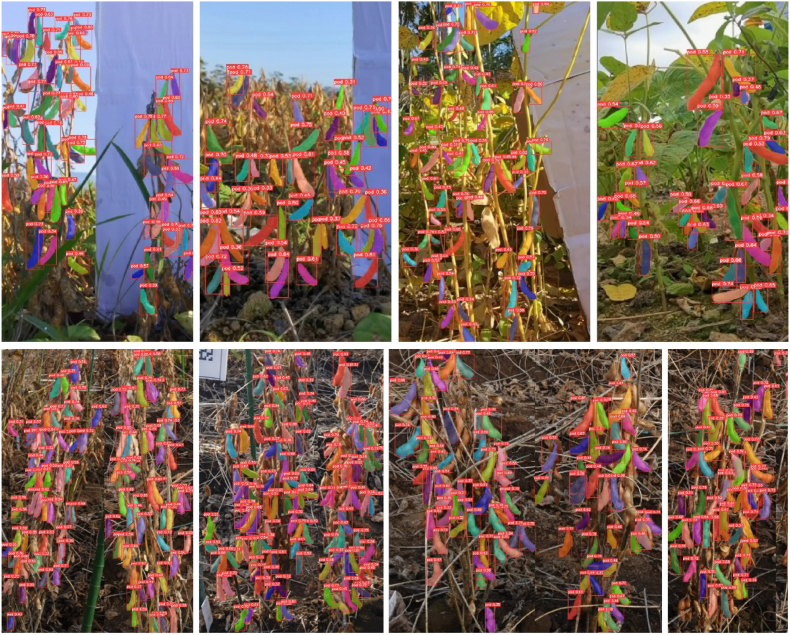
PodNet instance segmentation results for images without a backdrop. Top: Some images from the custom dataset. Down: Some images from the 2021 dataset [33].

The final PodNet model achieves an mAP@50 of 0.786, 2.5 ​% higher than that of the v8n-seg model. When the image resolution is set to 640, the threshold of the IoU is set to 0.5, and the confidence level is 0.3. The comparative results of the PodNet improvement are shown in Fig. 10 (a). PodNet can segment more pods in the root areas of soybean plants under complex backgrounds and poor lighting conditions. Moreover, it generates more realistic masks for pods that are occluded or traversed by branches and leaves. More visualization examples of pod instance segmentation on field soybean images are shown in Fig. 11, Fig. 12. Quantitative analysis and visualization results demonstrate that, after adopting the HPA strategy and U-EMA Protonet, PodNet is effective and robust for soybean plants of different cultivars and growth stages. However, segmentation failure issues persist in certain scenarios. For example, for severe disease damage pods in Fig. 10 (b), the highly similar textures between leaves and pods lead to undersegmentation of PodNet. This may be due to the limited number of soybean images with disease in the dataset. For complex occlusion scenarios, the model also experiences segmentation failures. These issues can be solved effectively by increasing the number of similar images and improving the precision of annotations.

Additionally, to test the correlation between the performance of PodNet and the absence of a backdrop, we collected a set of images from custom high-quality static images and single soybean plant images in the 2021 dataset [33]. In these images, the backdrop is partially or totally out of the field of view. As shown in Fig. 12, PodNet achieves competitive pod segmentation performance in preharvest fields with complex background conditions. The robust performance of PodNet on soybean images without a pure-color background can be attributed to two primary factors. First, the inherent capability of CNNs for semantic feature learning allows PodNet to focus on capturing essential characteristics of soybean pods rather than relying on background information. This enables the model to learn discriminative features that generalize well to images with different backgrounds or no background. Second, data augmentation techniques, including random cropping, rotation, flipping and combinations, were employed during training to simulate diverse environmental conditions. These augmentations help the model become less sensitive to background variations, further enhancing its ability to perform accurately on images with natural backgrounds. However, it has some limitations in handling distant small and cracked pods, which is predictable and consistent with the domain of the training set. Naturally, adding these backdrop-free images to the training set can effectively improve the model's performance for similar images. To a certain extent, the transfer learning ability of the PodNet model has been verified. These examples also underscore the necessity of the backdrop, which provides the feasibility of extracting soybean plants of the current row from multiplant images. Otherwise, the inability to distinguish the foreground row of plants in densely planted soybean fields would preclude subsequent cross-scale phenotypic analysis, such as pod counting per plant, seed counting per plant, plant height and branching structure.

Without employing TensorRT or ONNX conversion for inference acceleration, the average inference latency per image is 8.8 ​ms on an NVIDIA RTX 2070 GPU and 32.0 ​ms on the edge computing device Jetson AGX Orin, thus enabling real-time inference. Experiments confirm that the HPA and U-EMA strategies are effective extensions and optimizations of existing architectures, preserving the model's real-time performance while enhancing segmentation performance in complex scenes. The proposed methods contribute new model architecture design insights for single-stage instance segmentation methods.

## 4 Discussion

In general machine vision studies based on deep learning, the core issues are dataset construction and model development. Correspondingly, agricultural vision research primarily faces the challenges of high data acquisition costs and limited computational resources. Owing to the difficulty of collecting and annotating field-based soybean datasets, current studies on pods and other phenotypes are mostly limited to synthetic data and indoor environments. The proposed workflow for building the pod dataset offers feasible and relatively cheap guidance for building an in-field crop dataset. First, acquiring raw data through video modality leverages the photography capabilities of handheld smartphones, significantly reducing the labor intensity required for data collection while maximizing the capture of temporal information. Second, the use of automated frame extraction and no-reference image quality assessment methods to batch-process videos into high-quality static images requires minimal human intervention, greatly increasing the efficiency of field image acquisition. Finally, this paper validates the feasibility and effectiveness of employing advanced large visual models to assist data annotation, demonstrating a referenceable workflow for other agricultural perception tasks.

Based on the idea of prototype generation and mask coefficient combination, instance segmentation models have achieved real-time inference for the first time. PodNet improves the Protonet module before the prediction head. From the input and output perspective, the proposed HPA strategy applies the classic feature pyramid structure to the pathway of Protonet channel aggregation. Multiscale feature maps are reshaped to identical resolutions and fused into the mask prototype. The U-EMA adds upsampling and attention mechanisms to the FCN structure of the Protonet itself, transforming the computational graph of the entire network to be more consistent with the architecture of the U-shaped encoder-decoder. The proposed components and methods are validated through comprehensive ablation studies, which also provide reference practices for the design of instance segmentation model structures, especially the selection of upsampling operations at different stages of the model. To the best of our knowledge, PodNet is the first real-time pod instance segmentation model for preharvest fields.

For future work, there are areas that can be improved in the current research. For the PodNet model design, the channel contribution for merging three scale features into masks can be verified by more detailed experiments in the HPA strategy. With respect to the experiments and optimizations of the U-EMA module, we will continue to explore how to obtain more advantageous performance improvements with increased computational complexity. The current method, which relies solely on RGB images, is basically unable to handle occlusions caused by leaves, leading to a significant decrease in the model's applicability to soybean images with a high leaf density. Combining with other imaging devices or employing multiview sensing techniques might help address this issue. To enhance the poor segmentation performance of hard cases, we can adopt a method where the entire image is divided into batches and fed into the model for inference, with the results subsequently stitched together. Based on the current image dataset of whole soybean plants in fields, it is convenient to conduct further phenotypic research related to soybean stems, leaves, and seeds. Moreover, it is possible to further explore self-supervised learning based on the image reconstruction paradigm, thereby avoiding the need for extensive manual labeling efforts. Following the proposed workflow of dataset construction and model design, we plan to develop a multitask soybean field phenotyping system and deploy it on a ground-mobile platform for field robot inspection.

## 5 Conclusions

In this paper, we present a workflow that automatically selects high-quality images from video modality data and then uses large vision models to assist with pixel-level annotation, significantly reducing the difficulty and cost of constructing agricultural in-field visual datasets. Through this workflow, an instance segmentation dataset with 488 images and 20k soybean pod annotations is built. The first real-time field soybean pod instance segmentation model, PodNet, is developed based on YOLOv8 architecture. To solve the issues of small and dense pod perception, the HPA strategy is proposed to fuse multiscale feature maps to generate the mask prototype, and the U-EMA Protonet is designed by adding upsampling and attention methods to form a U-shaped decoder structure. On the custom dataset, PodNet achieves an mAP@50 of 0.786, representing a 2.5 ​% improvement over the baseline. The competitive performance on backdrop-free soybean images in preharvest fields provides a promising possibility for cross-scale phenotyping. These datasets and methodologies help pave the way for high-throughput field phenotyping, facilitating advancements in soybean breeding research and cultivation practices.

## Author contributions

S. Zhou: Methodology, Writing - original draft, Formal analysis.

Q. X. Sun: Funding acquisition, Data curation.

N. Zhang: Investigation, Writing - review & editing.

X. J. Chai: Conceptualization, Project administration.

T. Sun: Supervision, Resources.

## Data availability

The field soybean pod instance segmentation dataset is open sourced for the research community at https://github.com/Boatsure/PodNet.

## Funding

This work was funded in part by the 10.13039/501100002858China Postdoctoral Science Foundation [grant number NO.2023M743821]; the Beijing Smart Agriculture Innovation Consortium Project [grant number BAIC10-2024]; the ​Innovation Program of Chinese Academy of Agricultural Sciences [CAAS-ASTIP-2025-AII] ; the Central Public-interest Scientific Institution Basal Research Fund [No.JBYW-AII-2025-04].

## Declaration of competing interest

The authors declare that they have no known competing financial interests or personal relationships that could have appeared to influence the work reported in this paper.
