# Supplementary material for: PodNet: Pod real-time instance segmentation in pre-harvest soybean fields
Source: Plant Phenomics. 2025 May 19;7(2):100052. doi: 10.1016/j.plaphe.2025.100052 (PMC12710035; doi:10.1016/j.plaphe.2025.100052)
Supplement: Multimedia component 1 [file mmc1.pdf]

Supplementary Materials

A: NR-IQA definition

Table 4: No-reference image quality assessment algorithms.

NR-IQA	Equation	Caption
Image clarity	$C = \mathrm{Var}\left(\nabla^2 I\right)$	∇^2 -Laplacian operator; I -Image; Var -Variance calculation.
Edge sharpness	$S = \frac{1}{N} \sum_{i=1}^{N} \sqrt{\left(\frac{\partial I}{\partial x}\right)^2 + \left(\frac{\partial I}{\partial y}\right)^2}$	$\frac{\partial I}{\partial x}$ -Sobel gradient in the x direction; N -Pixel number.
Image entropy	$E = -\sum_{i=1}^{256} p_i \log_2 (p_i)$	p_i -Probability of a pixel having gray level i.
GLCM energy	$G = -\sum_{i=0}^{N-1} \sum_{j=0}^{N-1} P(i,j)^2$	$P(i,j)$ -Probability of gray levels of adjacent pixels being i and j; N-Number of gray levels.

B: Model training setting

Table 5: Model training hyperparameters settings.

resolution	optimizer	batch size	IoU	learning rate	momentum	weight decay	mosaic	scale
640	Adam	8	0.4	0.01	0.937	5e-4	0.3	0.2

Table 6: Evaluation metrics and their formulas in pod instance segmentation task.

**Name**	**Formula**	**Explanation**
Accuracy	$\text{Accuracy} = \frac{\text{TP+TN}}{\text{TP+TN+FP+FN}}$	The ratio of correctly predicted samples (both positive and negative) to the total number of samples.
Precision	$\text{Precision} = \frac{\text{TP}}{\text{TP+FP}}$	The proportion of true positives among all predicted positives.
Recall	$\text{Recall} = \frac{\text{TP}}{\text{TP+FN}}$	The proportion of true positives among all actual positives. Measures the model's ability to identify all positive samples.
IoU	$\text{IoU} = \frac{\text{Area of Overlap}}{\text{Area of Union}}$	The ratio of the intersection area to the union area between the predicted mask and the ground truth mask. A core metric for evaluating localization accuracy, used as a criterion for correct prediction.
AP	$\text{AP} = \int_0^1 P(R)\, dR$	Average Precision is the area under the Precision-Recall curve, representing the average Precision across different Recall levels. Used to evaluate prediction performance for a single class.
mAP@50	$\text{mAP@50} = \frac{1}{N} \sum_{i=1}^{N} \text{AP@50}_i$	The mean of AP across all classes, calculated at an IoU threshold of 0.5. In this paper, there is only one class pod.
mAP@50-95	$\text{mAP@50-95} = \frac{1}{10} \sum_{j=1}^{10} \text{mAP@IoU}_j$	The mean of mAP values calculated across multiple IoU thresholds ranging from 0.5 to 0.95 (with a step size of 0.05). Provides a comprehensive evaluation of model performance across varying IoU thresholds.
